# Whole-Genome Sequence, Assembly and Annotation of an Invasive Plant, *Lonicera maackii* (Amur Honeysuckle)

**DOI:** 10.3390/plants11233253

**Published:** 2022-11-26

**Authors:** Erin Kesel, André O. Hudson, Michael V. Osier

**Affiliations:** Thomas H. Gosnell School of Life Sciences, Rochester Institute of Technology, 1 Lomb Memorial Drive, Rochester, NY 14623, USA

**Keywords:** *Lonicera maackii* honeysuckle, invasive species, genome, assembly, EPSP synthase

## Abstract

The invasive species *Lonicera maackii* (Amur Honeysuckle) is an increasing problem sweeping from the eastern United States toward the west, impacting normal forest development and animal survival across multiple taxa. Little is known about the genomics of this species, although a related invasive, *Lonicera japonica*, has been sequenced. Understanding the genomic foundation of the *Lonicera maackii* species could help us understand the biochemistry and life history that are the underpinnings of invasive success, as well as potential vulnerabilities and strengths which could guide research and development to control its spread. Here we present a draft, but high-quality, short-read whole-genome sequence, assembly, and annotation of *Lonicera maackii*, demonstrating that inexpensive and rapid short-read technologies can be successfully used in invasive species research. Despite being a short-read assembly, the genome length (7.93 × 10^8^) and completeness (estimated as 90.2–92.1% by BUSCO and Merqury) are close to the previously published chromosome-level sequencing of *L. japonica*. No bias, by means of a Gene Ontology analysis, was identified among missing BUSCOs. A duplication of the 5-enolpyruvylshikimate-3-phosphate (EPSP) synthase gene in both *Lonicera* species is identified, and the potential impact on controlling these invasive species is discussed. Future prospects for a diversity analysis of invasive species is also discussed.

## 1. Introduction

Invasive species are one of the biggest threats to biodiversity across the world. It has been estimated that in the United States alone, the annual economic and environmental cost of invasive species is at least $120 billion dollars [1]. One invasive species, *Lonicera maackii* (Rupr.) Herder (Caprifoliaceae), an invasive bush honeysuckle, has caused major problems in many parts of the eastern and central United States. While the economic impact of *L. maackii* has not been quantified, this species has been shown to be capable of causing significant environmental damage in the areas it has invaded [2,3,4].

*L. maackii*, commonly known as Amur Honeysuckle or Maack’s honeysuckle, was originally brought to North America from Europe and Asia in the 1800s as an ornamental shrub for its flowers and fruit [5]. *L. maackii* is an upright, multi-stemmed shrub that can grow up to six meters tall, spread nine meters across, and has stems with diameters up to fifteen centimeters [6]. The shrub has dark green, acuminate leaves. In early spring, the plant produces white to pink paired axillary flowers that fade to dark yellow with age, and in the fall, the shrub’s small, glossy red or orange berries ripen [6]. *L. maackii* is a diploid organism with a chromosome count of 2n = 18 [7].

In the 1950s, *L. maackii* began to escape cultivation in the United States [8]. Since its initial escape, *L. maackii* has spread to many parts of the eastern and central United States and parts of Canada [9]. Invasion of *L. maackii* has caused decreased species richness in invaded areas [10,11,12,13,14]. Species richness under the crowns of *L. maackii* bushes has been found to be lower than under native shrubs. Moreover, there has been reduced species richness in forests invaded by *L. maackii*, and reduced tree seedling density in invaded forests [10]. *L. maackii* has also been shown to cause reduced growth and reproduction of native plant species, affecting the native production of fruit, seeds, and flowers [15]. Furthermore, *L. maackii* invasion can negatively affect animals in the invaded areas. In a study by Watling et al. [13], forest plots invaded by *L. maackii* had lower species richness and evenness of amphibian species compared with plots without *L. maackii*. The mean maximum daily temperature was significantly lower in the plots invaded by *L. maackii* than in the uninvaded plots, demonstrating that *L. maackii* invasion causes changes to the microclimate of invaded areas.

One key to *L. maackii*’s success as an invasive species is its means of reproduction. *L. maackii* reproduces via seed dispersal. Native animals, including birds and deer, consume the bush’s berries and disperse the seeds [16,17]. *L. maackii* is found along the paths followed by migratory birds during migration season. During fall migration, *L. maackii* berries are accessible to migratory birds as they travel south. However, the nutritional value of *L. maackii* berries has been shown to be inferior to that of native shrubs, having lower fat content and lower energy density than berries of native species [18]. Due to its invasion, *L. maackii* berries are increasingly more available than the more nutritious native berries, so migratory birds are forced to consume the less nutritious berries. The consumption by the migratory birds also amplifies the spread of *L. maackii* because seed dispersal occurs while the birds continue along their migration path. Additionally, because the birds must consume more berries for equal energy value, the number of seeds dispersed is even greater.

Next-generation sequencing technologies allow scientists to determine genomic sequences quickly and inexpensively. An advantage to using next generation sequencing to understand invasives is that it allows scientists to use a well-established technique to study the underlying molecular mechanisms of why invasives are so successful in their non-native environments. For example, the Asian Longhorned beetle, an invasive insect, eats and destroys native tree species. By sequencing the genome of the Asian Longhorned beetle, ref. [19] were able to identify genes encoding enzymes that enable the beetle to degrade the native trees, including enzymes that degrade polysaccharides in plant cell walls.

Currently, there is only one publicly available assembled genomic sequence for any *Lonicera* species [20]. Determining the genomic sequence of multiple invasive *Lonicera* species, such as *L. maackii,* will help to identify key genes that may aid in the understanding of these invasive species and provide insight into the best control mechanisms. As differentiating *Lonicera* species is difficult by visual characteristics, having an available genome sequence for *L. maackii* is important to early identification of *L. maackii*. Genomic evidence could also aid in the identification of young *L. maackii* plants from other plants in an area so that they can be removed before they reach reproductive age and are too large to be easily removed.

One gene of relevance to the chemical control methods primarily used on *L. maackii* is the 5-enolpyruvylshikimate-3-phosphate (EPSP) synthase gene. In some weedy plant species, resistance to glyphosate, a common herbicide, has been associated with tandem duplications of the EPSP synthase gene [21]. The mechanism of resistance by the duplications is unresolved [21]. However, tandem duplications of the same gene have been associated with glyphosate resistance in at least eight plant species. The evidence suggests these duplication events were the product of convergent evolution [21]. Given the importance of glyphosate to the control of *L. maackii* and other invasive honeysuckles, an exploration of these genes is warranted.

Here, we describe the assembly and annotation of a draft genome sequencing of *L. maackii*. The assembly presented is both highly complete and contiguous. In a search of both the *L. maackii* and *L. japonica* genomes, it was identified that there are two copies of the EPSP synthase gene. These *Lonicera* EPSP synthase genes are suggestive of a duplication of the gene in a common ancestor. These results suggest that it would be beneficial for future studies to assess if EPSP synthase inhibitors may not be a good long-term control method.

## 2. Results

### 2.1. Sample Identification

**Figure 1:**
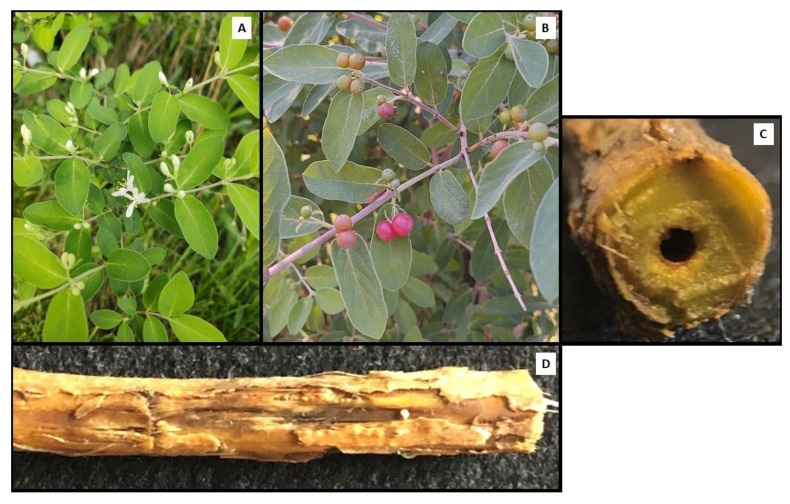
Images of the sequenced plant. (**A**) Leaves and flowers. (**B**) Opposite leaf pattern along stem and berries. (**C**) Transverse section of stem showing hollow structure. (**D**) Longitudinal section of stem.

The physical characteristics of this particular plant (Figure 1) were used to confirm identification. The shape of the leaves was elliptical with long and narrow points, with pairs positioned opposite of each other along the stem. The berries were red, round, and positioned at leaf junctions along the stem. The flowers were white and in pairs with the characteristic *Lonicera* shape. In addition, characteristic of invasive *Lonicera* species, the stem was observed to be hollow. All the observed characteristics were consistent with an identification as a *Lonicera* species, specifically *Lonicera maackii*.

### 2.2. Sequencing Data Quality

Raw sequencing data were submitted to the Sequence Read Archive (BioProject PRJNA521295). The sequence data had high-quality scores [22] and consistency in length due to truncation by BGI. Neither mtDNA nor cpDNA was filtered previous to sequencing. Using alignment methods, 0.7% (2616644/363819070) and 15.3% (71421541/464431676) of reads were mtDNA and cpDNA, respectively. It should be noted that the chloroplast genome used for filtering was from the same species, but that the mitochondrial genome was from *Helianthus annuus*. At the time of the analysis, *H. annuus* was the most closely related available genome to *L. maackii*, with both being campanulids. As a result, mitochondrial genome sequences may not have all been filtered out. These non-genomic reads were removed previous to assembly. No other quality control was performed previous to assembly, as per the instructions for the assembler. Additionally, any mtDNA incorporated into the assembly was automatically filtered out by NCBI during the genome submission process.

### 2.3. Assembly Contiguity and Completeness

The genome sequence was submitted to NCBI (accession JAMFLW000000000). Genome contiguity (Table 1) was sufficient to identify key genes. Although the number of contigs was great, the L50 was sufficiently small, with a large N50, to represent many genes with little or no fragmentation. The total length of contigs for the assembly (7.93 × 10^8^) is close to the predicted genome length of *L. japonica* (8.43 × 10^8^).

Indeed, a BUSCO analysis revealed that both the assembly (92.1%) and alignment (90.9%) identified most of the conserved genes from eudicot_odb10. However, both methods had a non-negligible number of genes not present in the other (Table 2). Of the 2178 BUSCOs identified in the alignment, 133 (6.1%) were not found in the assembly. Likewise, of the 2153 BUSCOs identified in the assembly, 108 (5.0%) were not found in the alignment. Combined, 40 BUSCOs were not found by either method, for 98.3% completeness. The assembly had more fragmented and duplicated BUSCOs than the alignment did. However, both had a high degree of complete BUSCOs (81.4% and 86.9%, respectively, for the alignment and assembly), demonstrating acceptable contiguity for a draft genome.

Additionally, an analysis with Merqury [23] was conducted. Merqury estimated 90.17% k-mer completeness, similar to the BUSCO analysis. It also estimated a QV of 40.73, which corresponds to >99.99% accuracy for consensus-based calls [23]. A copy number spectrum was constructed with Merqury. Assembly k-mers absent from the read set had totals that were lower than 1.5 × 10^6^ (Supplementary Figure S1), in agreement with a low assembly error rate per Rhie et al. [23]. Two peaks were observed, which is in agreement with the expectations of an unphased assembly. Very few 2-copy k-mers were observed three times (green), indicating a good k-mer completeness per Rhie et al. [23].

The assembly had a 36.5% GC content, in line with the 34.5% observed previously in *L. japonica* (NCBI ASM2146441v1). BLAST annotation identified 42,348 *H. annuus* genes, including duplications of those genes. This is somewhat similar to the 33,939 genes identified by Pu et al. [20] in *L. japonica* through expressed transcripts.

In comparing the previously published *L. japonica* genome, 79,206 indels and 627,273 single nucleotide substitutions were identified. Of these single nucleotide substitutions, the transition/transversion ratio was 1.73, which is within expectations for two very closely related species and a quality alignment [24]. For both *L. japonica* and *L. maackii*, multiple BUSCOs are missing. Cross-referencing the missing BUSCOs with associated Gene Ontology terms reveals only a handful of categories (Table 3). Most have only a small number of missing BUSCOs, suggestive of relatively minor portions of the genome that were not sequenced. The exception would be GO:0016020 (Membrane), which is a second-tier Cellular Location term and not very informative.

### 2.4. EPSP Synthase

**Figure 2:**
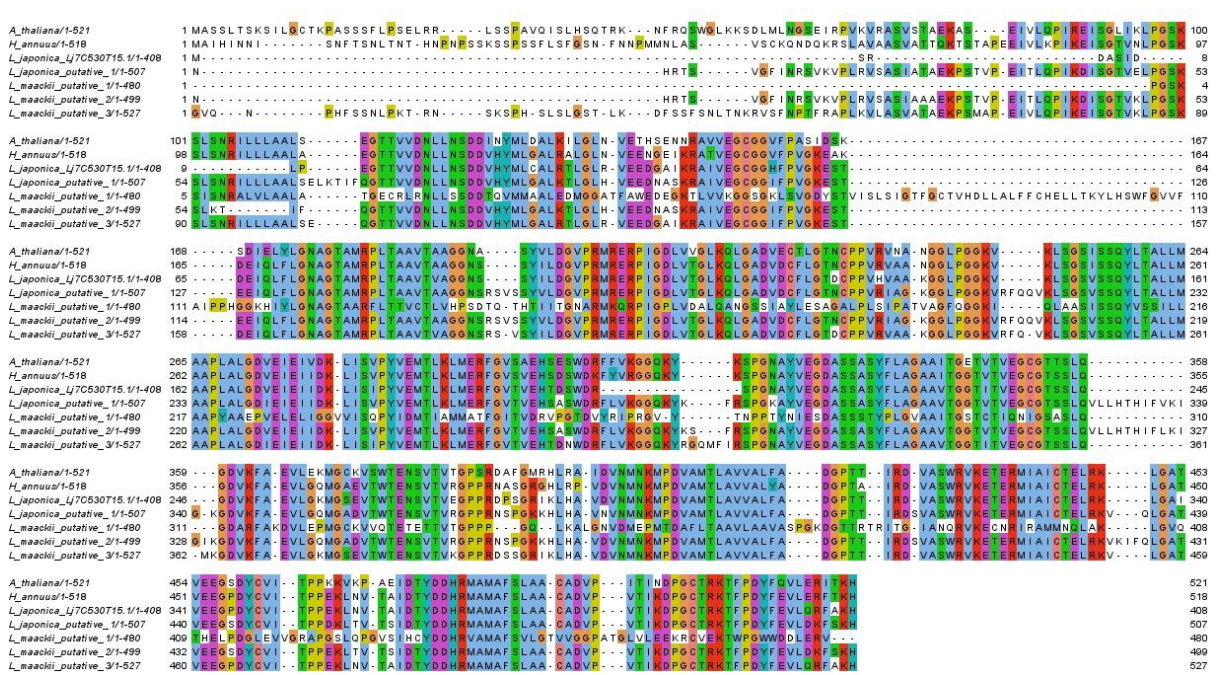
Alignment of known and predicted EPSP synthase protein sequences. Sequences are identified by species name. Proteins predicted through sequence homology are named as “putative”.

Two potential EPSP synthase genes were identified in both *L. maackii* (L_maackii_putative_2 and L_maackii_putative_3) and *L. japonica* (L_japonica_Lj7C530T15.1/408 and L_japonica_putative_1) by homology with the *A. thaliana* EPSP synthase (Figure 2). One of the *L. japonica* genes was annotated previously by [20] on chromosome 7 (Lj7C530T15.1). The other predicted *L. japonica* EPSP synthase is located on chromosome 9 (56844721–56852241). A third potential homolog was identified in *L. maackii*.

**Figure 3:**
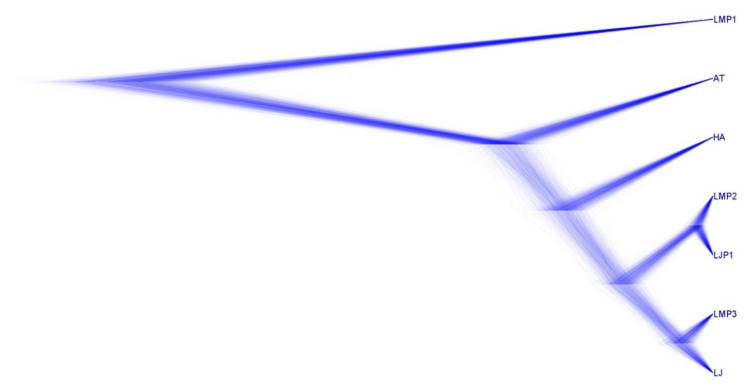
Maximum likelihood tree for predicted protein sequences. Derived species are identified by abbreviation (AT—*A. thaliana*, HA—*H. annuus*, LM—*L. maackii*, and LJ—*L. japonica*). “P” annotates protein sequences predicted by sequence homology.

However, (L_maackii_putative_1) appears to have diverged previous to EPSP synthase (LMP1 in Figure 3). As a result, LMP1 serves as an outgroup to the EPSP synthase genes. *H. annuus* EPSP synthase is included as a more recent ancestor to the *Lonicera* taxa for comparison. The other *Lonicera* sequences (LMP2 and LMP3, respectively, for *L. maackii*, and LJP and LJP1 for *L. japonica*) form two pairs of near-perfect identity with each other (Figure 3), suggesting a possible duplication in a common ancestor after *Lonicera* diverged from *H. annuus*.

## 3. Discussion

Here is announced a new *Lonicera* genome sequence. The assembly has acceptable completeness as evidenced by BUSCO and Merqury. Further sequencing with long-read technology would be beneficial to resolving the chromosomal structure of this specific species of *Lonicera*. Having a draft genome for this species, which is invasive in the eastern United States, will aid studies of the potential effects of this species on native species and potential novel vulnerabilities that can be leveraged to control this invasive plant, as has been observed in other studies [25]. Typically, glyphosate is routinely and successfully used to control this species. However, it is well established that resistance to glyphosate in undesired plants is a growing problem [26].

Indeed, it is concerning that we were able to identify two potential copies of the EPSP synthase gene, known for resistance to glyphosate, through duplications of the gene [21]. As noted by Pu et al. [20], there is evidence of a genome duplication event in the evolutionary past of L. japonica. Interestingly, Bowers et al. [27] recently proposed a model explaining the correlation between lower GC content, such as observed in the two *Lonicera* species discussed here, and in species with recent duplications. In *L. japonica*, these two copies of EPSP synthase genes were indeed located on two different chromosomes. Although this short-read study was unable to uniquely identify the chromosomes of the *L. maackii* EPSP synthase genes, the significant sequence differences suggest that these are indeed remnants of an ancestral duplication. It would also be interesting to see if the duplication is observed in the *L. japonica* sequencing by Yu et al. [28] which was published subsequent to our analysis. Studies of the transcriptional activity of both genes, in both *Lonicera* species, would be important to assess the potential for duplications of these genes to instill resistance. If both copies are transcribed and active in glyphosate resistance, *Lonicera* could be an unusual and problematic invasive taxa. Alternatively, one copy in each species may be a pseudogene, or perhaps the functions of these copies may have diverged, as evidenced by their 82.8% identity at the protein level. Most differences are outside of key domains (Figure 1), indicating that enzyme kinetic studies may be necessary to determine the potential impact of these genes. It would also be important to examine these genes in other *Lonicera* species, as well as closely related species, to identify the extent of this duplication and its potential impact in other related invasives.

Sequencing of native *Lonicera* species may also allow us to identify genomic differences which could be leveraged to control the invasive species. In New York State alone, at least four species of honeysuckle are classified as invasive: *L. maackii*, *L. morrowii, L. tatarica*, and *L. japonica*. Honeysuckle is a growing invasive species problem, and there is ripe territory for the identification of novel mechanisms to control invasive plant species. Given that all invasive honeysuckles in this region have hollow stems, while native species do not, identifying the underlying genetic causes for this physiological difference and understanding its mechanisms and evolutionary purpose might be fruitful. Utilization of less expensive short-read assemblies and alignments could speed the research of these species, and other terrestrial invasive plants. For example, more of the invasive honeysuckle species need to be sequenced in order to compare the similarities and differences that lead to invasive success.

Finally, the genetic diversity of these species is unknown. Diversity of the EPSP synthase gene(s) could also contribute to resistance to glyphosate, as well as other survival tactics the species may use to make them more effective invasive plants. It has been established that invasive honeysuckles can hybridize [29], whereby causing changes in gene expression, including hybrid vigor. Such hybridization compounds the difficulty of quantifying genetic diversity in these species. The degree of genetic diversity of EPSP synthase, as well as other genes related to control resistance and invasive success, will be critical in assessing the potential for evolving resistance to control due to coding variation or changes in the expression of the gene. Indeed, Yu et al. [30] identified a double amino acid substitution in *Eleusine indica* which results in a >180-fold increase in glyphosate resistance. For non-crop weeds, invasive honeysuckles could create a unique challenge. Inexpensive short-read technologies will enhance our ability to collect sufficient data to assess population and gene-level genetic diversity, degrees of hybridization, and potential for resistance to control mechanisms.

## 4. Materials and Methods

### 4.1. Sample Preparation and Sequencing

Three samples were taken from the leaves of a bush honeysuckle plant on a private property located in Bristol, Western New York. Genomic DNA was isolated from the three samples using the Qiagen DNeasy Plant Mini Kit. After arriving to the BGI (Cambridge, MA, USA) sequencing facility, a Qubit fluorometer and the DNA BR kit were used to determine the concentration of DNA in each sample. Sample integrity was determined by gel electrophoresis with 1% agarose gel run at 150 V for 40 min. The concentration of the three samples was below that required for sequencing, so the three samples were pooled into one. The pooled sample was sequenced using the DNBseq platform at BGI, using paired-end 100 base-pair reads, as an inexpensive and rapid method.

### 4.2. Quality Control

FastQC version 11.9 [31] was used to evaluate the quality of the raw data obtained from BGI. Reads likely belonging to either the mitochondrial or chloroplast genomes were removed. The raw reads were aligned to the chloroplast reference genome sequence for *L. maackii* (GenBank MN256451.1) using STAR [32], allowing for at most two mismatches. Reads that did not map to the chloroplast were then aligned to the mitochondrial reference genome sequence for *Helianthus annuus*, common sunflower (GenBank NC_023337.1), again using STAR and allowing for up to two mismatches. No reference sequence was available for the *L. maackii* mitochondrial genome, so the most closely related mitochondrial reference sequence was used. Reads that did not map to the chloroplast or the mitochondrial reference genomes were written to new FASTQ files that were used for assembly.

### 4.3. Genome Assembly and Alignment

MaSuRCA [33] has been shown to produce high-quality genome assemblies and was used for this de novo genome assembly [34]. Raw reads with chloroplast and mitochondrial reads removed were supplied to the assembler. No further quality control is recommended by the authors of MaSuRCA. The JELLYFISH_HASH_SIZE parameter was set to 60,000,000,000, which was 20× the originally approximated genome size of 3 billion bases. Parameter USE_LINKING_MATES was set to 1. All other parameters were kept at default values.

Raw reads were also aligned to the *L. japonica* reference genome [20] using STAR Aligner [32] with default parameters.

### 4.4. Assembly and Alignment Evaluation

Contiguity was evaluated using the abyss-fac function from the command-line tool ABySS [35]. The estimated genome size was required as an input parameter for this program. Genome size was estimated using the genome sizes of relatives as defined in the literature. Both *L. japonica* and *L. maackii* have a diploid chromosome count of 2n = 18 [36]. The genome of *L. japonica* has been estimated to have a 1Cx value of 1135 Mbp. To approximate the genome size of *L. maackii* for abyss-fac, the 1Cx value of *L. japonica* was used, and the total diploid genome size for *L. maackii* was estimated to be 2270 Mbp. Assembly and alignment completeness was evaluated using BUSCO [37] with the eudicot_odb10 lineage.

### 4.5. Genome Annotation

Genome annotation was performed with a local tBLASTn (BLAST+ 2.5.0) using the proteome of *Helianthus annuus* (UniProt proteome ID UP000215914) as a reference. The BLOSUM62 scoring matrix was used with a gap opening penalty of 11 and a gap extension penalty of 1. Matches with e-values greater than 0.00001 or an alignment length of less than 50 residues were removed. The results were then converted to a GFF format using a custom script. Gene Ontology terms associated with BUSCOs were mapped using OrthoDB 10v1 [38].

### 4.6. Identification of EPSP Synthase Homologs

Putative EPSP synthase genes in the two *Lonicera* species were identified with a local tBLASTn (BLAST+ 2.5.0) using the *A. thaliana* EPSP synthase protein sequence (AT1G48860.1) as a query. The *H. annuus* EPSP synthase (XP_022017499.1) was identified in the literature [39] and confirmed with tBLASTn against the *H. annuus* genome in the same manner as the two *Lonicera* species. Protein sequences were extracted from the BLAST results and aligned by T-Coffee [40]. A phylogenetic tree was generated using MEGA version 11 [41] for alignment. The MEGA results were imported into the BEAUti algorithm of BEAST2 [42], followed by performing greater than 1 × 10^6^ samples with BEAST2. The final tree was plotted using DensiTree from BEAST2. Default parameters were used throughout the tree generation.

## Figures and Tables

**Table 1:** Contiguity statistics. Standard metrics of assembly contiguity were generated using ABySS.

**Number of contigs**	205,041
**L50**	12,727
**LG50**	205,041
**N80**	2729
**N50**	15,582
**N20**	39,209
**E-size**	22,482
**Maximum contig length**	161,169
**Minimum contig length**	103
**Sum of contig lengths**	7.93 × 10^8^

**Table 2:** Comparison of BUSCO analysis on de novo assembly and alignment against the *L. japonica* reference sequence. Counts represent the number of individual BUSCOs that met the respective criteria in the assembly and alignment.

		Assembly				
		Complete	Fragmented	Duplicated	Missing	Total
**Alignment**	**Complete**	1704	133	57	128	2022
	**Fragmented**	54	8	0	2	64
	**Duplicated**	39	3	47	3	92
	**Missing**	96	9	3	40	148
	**Total**	1893	153	107	173	2326

**Table 3:** Gene Ontology categories for missing BUSCOs in both the previously published *L. japonica* and the current *L. maackii* genomes.

	*L. japonica*	*L. maackii*
GO:0000413: protein peptidyl-prolyl isomerization	0	2
GO:0002098: tRNA wobble uridine modification	0	3
GO:0005622: intracellular anatomical structure	0	3
GO:0005634: nucleus	0	4
GO:0005739: mitochondrion	2	2
GO:0006265: DNA topological change	0	2
GO:0006281: DNA repair	0	2
GO:0006468: protein phosphorylation	4	3
GO:0006508: proteolysis	6	8
GO:0006629: lipid metabolic process	0	2
GO:0006777: Mo-molybdopterin cofactor biosynthetic process	0	2
GO:0007018: microtubule-based movement	0	2
GO:0007275: multicellular organism development	0	3
GO:0009507: chloroplast	7	5
GO:0009658: chloroplast organization	0	4
GO:0016020: membrane	17	11
GO:0016310: phosphorylation	2	2
GO:0032259: methylation	6	3
GO:0055085: transmembrane transport	3	3

## Data Availability

Raw sequencing data were submitted to the Sequence Read Archive (BioProject PRJNA521295). The genome sequence was submitted to NCBI (accession JAMFLW000000000).

## Supplementary Materials

The following supporting information can be downloaded at: https://www.mdpi.com/article/10.3390/plants11233253/s1, Figure S1: Merqury copy number spectrum.
