# Supplementary figures and images for: Whole-Genome Sequence, Assembly and Annotation of an Invasive Plant, Lonicera maackii (Amur Honeysuckle)
Source: Plants (Basel). 2022 Nov 26;11(23):3253. doi: 10.3390/plants11233253 (PMC9740181; doi:10.3390/plants11233253)

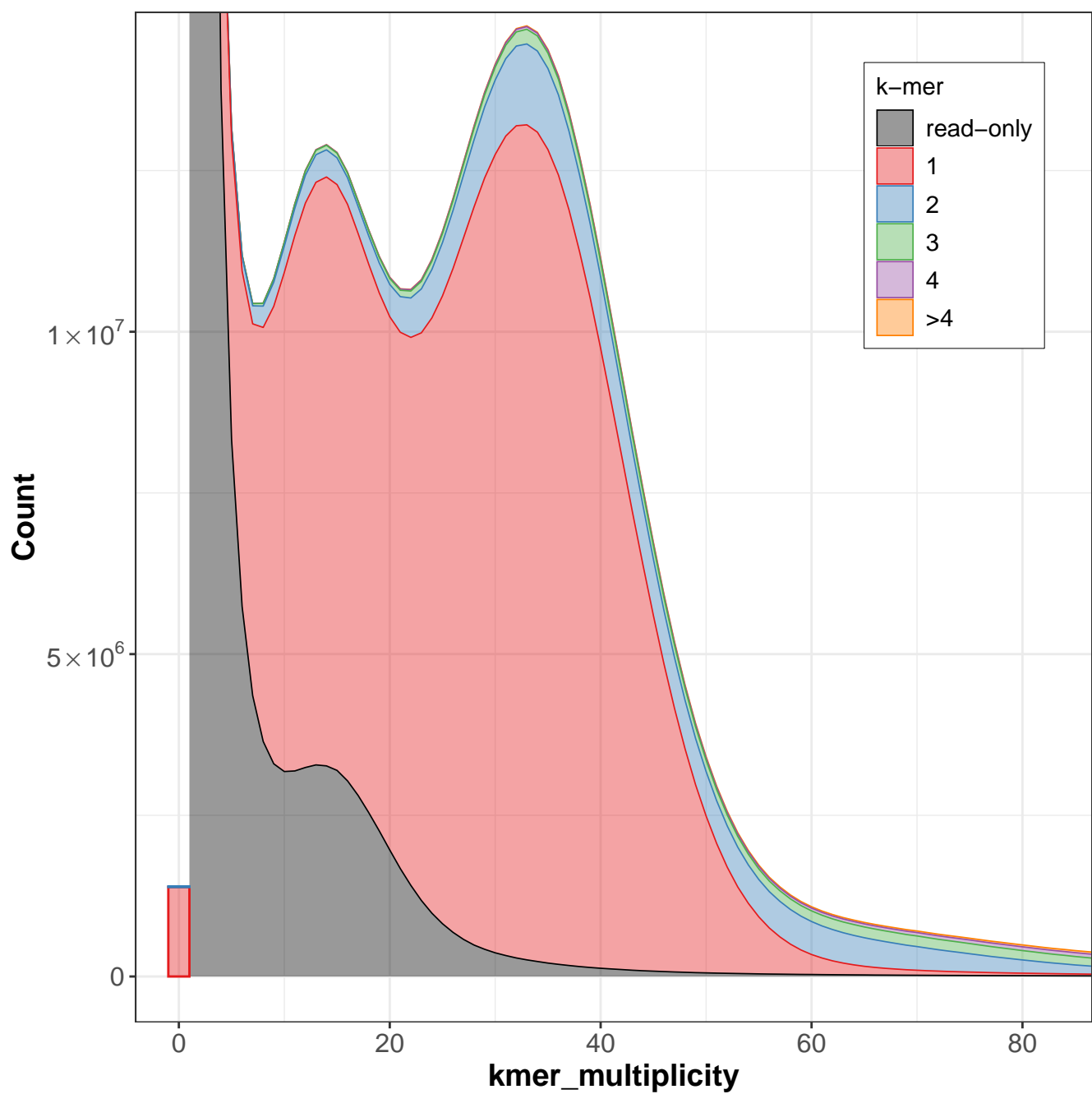

Supplement: Supplementary file 1 [file plants-11-03253-s001.zip › plants-1952331-supplementary.pdf]
